# Municipal Leachate Treatment by Fenton Process: Effect of Some Variable and Kinetics

**DOI:** 10.1155/2013/169682

**Published:** 2013-06-06

**Authors:** Mohammad Ahmadian, Sohyla Reshadat, Nader Yousefi, Seyed Hamed Mirhossieni, Mohammad Reza Zare, Seyed Ramin Ghasemi, Nader Rajabi Gilan, Razieh Khamutian, Ali Fatehizadeh

**Affiliations:** ^1^Social Development & Health Promotion Research Center, Kermanshah University of Medical Sciences, Kermanshah, Iran; ^2^Department of Environmental Health Engineering, School of Public Health, Tehran University of Medical Sciences, Tehran, Iran; ^3^Environment Research Center, Isfahan University of Medical Sciences (IUMS), Isfahan, Iran; ^4^Department of Environmental Health Engineering, School of Health, IUMS, Isfahan, Iran; ^5^Faculty of Public Health, Kermanshah University of Medical Sciences, Kermanshah, Iran

## Abstract

Due to complex composition of leachate, the comprehensive leachate treatment methods have been not demonstrated. Moreover, the improper management of leachate can lead to many environmental problems. The aim of this study was application of Fenton process for decreasing the major pollutants of landfill leachate on Kermanshah city. The leachate was collected from Kermanshah landfill site and treated by Fenton process. The effect of various parameters including solution pH, Fe^2+^ and H_2_O_2_ dosage, Fe^2+^/H_2_O_2_ molar ratio, and reaction time was investigated. The result showed that with increasing Fe^2+^ and H_2_O_2_ dosage, Fe^2+^/H_2_O_2_ molar ratio, and reaction time, the COD, TOC, TSS, and color removal increased. The maximum COD, TOC, TSS, and color removal were obtained at low pH (pH: 3). The kinetic data were analyzed in term of zero-order, first-order, and second-order expressions. First-order kinetic model described the removal of COD, TOC, TSS, and color from leachate better than two other kinetic models. In spite of extremely difficulty of leachate treatment, the previous results seem rather encouraging on the application of Fenton's oxidation.

## 1. Introduction

In the past decades, industrial growth and technology development lead to the increasing solid waste production [1]. According to the World Health Organization studies, in the many countries such as France, Canada, America, Norway, England, Spain, and Italy, sanitary landfilling had been recognized as the common, economic and acceptable method for solid waste disposal [2–4]. Landfill leachate is a complex waste matter, which usually causes adverse effects in the environment [5, 6]. The leachate characteristics are depended on the type of solid waste, soil properties, rainfall patterns, and age of landfill site. The concentration of nonbiodegradable and resistant materials with high molecular weight such as humic and fulvic acid arises with increase of the landfill site age [7].

Due to variable characteristic of landfill leachate, the coherent method for leachate treatment was not developed [8]. The concerning of solid waste leachate may be related to presence of heavy metals and nonbiodegradable organic materials in solid waste leachate and its adverse effects on human and the environment [3]. Application of biological treatment processes (as dominant treatment) cannot remove nonbiodegradable organic materials and additional treatments are required for leachate treatment [9]. Biological treatment processes are suitable for fresh leachate with high ratio of BOD_5_/COD but cannot be applied in the treatment of aged leachate in which biological treatment occurred, and ratio of BOD_5_/COD is low [10].

Chemical treatment methods based on production of hydroxyl radical (OH^•^) are known as advanced oxidation processes (AOP_s_) [11, 12]. Oxidation of organic compounds by Fenton solution called the Fenton reaction which is one of the advanced oxidation processes [13]. Fenton reaction is able to destroyed large number of organic compounds without producing toxic byproducts. Another main advantage of Fenton process is that the oxidation and flocculation occur simultaneously that result in removing more organic matters [14].

Fenton process is done according to the following mechanism [3]:

1. Fe^2+^ + H_2_O_2_ → Fe^3+^ + ^•^OH + OH^−^,
2. Fe^2+^ + H_2_O_2_ → Fe^2+^ + HO_2_^•^ + H^+^,
^•^OH + H_2_O_2_ → HO_2_^•^ + H_2_O,
^•^OH + Fe^2+^ → OH^−^ + Fe^3+^,
5. Fe^3+^ + HO_2_^•^ → Fe^2+^ + O_2_H^+^,
6. Fe^2+^ + HO_2_^•^ + H^+^ → Fe^3+^ + H_2_O_2_,
7. 2OH_2_^•^→ H_2_O_2_ + O_2_.

Oxidation potential of hydroxyl radical (OH^•^) is greater than ozone, one of the strongest oxidizing materials [15]. Hydrogen peroxide alone is not a strong factor in oxygen transfer, and oxidation of organic materials is better performed in the presence of Fe^2+^ ions [16]. The most important role of Fenton process is removing refractory and toxic organic compounds and increasing the degradability of resistant organic compounds. Fenton processes have been successfully used for treatment of the slaughterhouse, food, olive oil wastewater, and 2,4,6-trichlorophenol from industrial wastewater and landfill leachate [15, 17–22]. This method compared to other advanced oxidation processes is relatively less expensive and requires less time [23]. 

The aim of this study was to investigate the effect of Fenton process for solid waste leachate treatment from Kermanshah landfill (Iran). 

## 2. Experimental 

### 2.1. Leachate Characteristics

The Leachate sample was collected from the municipal solid waste landfill in Kermanshah city (Iran). The main characteristics of leachate were summarized in Table 1.

### 2.2. Chemicals

All chemicals were of analytical grade (Merck, Germany), and doubly distilled water was used throughout this study. The hydrogen peroxide (35% w/w) and ferrous sulphate (FeSO_4_·7H_2_O) were used for all experiment. The FeSO_4_ solution was prepared daily. H_2_SO_4_ and NaOH (1 N) were used for pH adjustment.

### 2.3. Experimental Procedure

In this study, the effect of Fenton process of leachate treatment was conducted in environment temperature in glass reactor using jar apparatus as a batch reactor. At first, rapid mix (120 rpm) was applied for 30 s, and then mixers were adjusted for 80 rpm for long time. The effect of various parameters including solution pH, Fe^2+^ and H_2_O_2_ concentrations, Fe^2+^/H_2_O_2_ molar ratio, and reaction time was investigated. All experiments were carried out with 1 L beakers, and the beakers were charged with 500 mL leachate [24]. 

### 2.4. Analytical Methods

The amounts of pH, COD, TOC, TSS, color, and alkalinity were analyzed in the laboratory by following the standard methods [25]. The pH, TOC, color, and alkalinity measurements were performed by using the WTW Multiparameter 340i, Shimadzu model TOC-CSH, spectrophotometer, and titration method, respectively. Closed reflux colorimetric method was used for COD analysis. For COD measurement of the supernatant, supernatant was put in the water bath at 50°C for 30 min for removal of remaining H_2_O_2_ in Fenton process experiments [3]. The removal efficiency was calculated by the following equations:

(1) $$\begin{array}{c} R=\left[\frac{C_{0}-C_{e}}{C_{0}}\right]\times 100. \end{array}$$

## 3. Results

### 3.1. Effect of Solution pH

**Figure 1:**
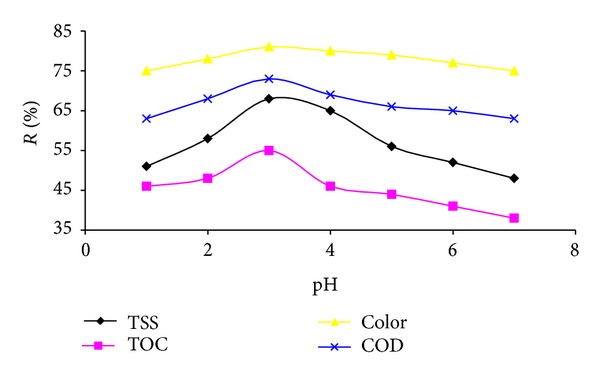
Influence of solution pH on COD, TOC, TSS, and color removal (H_2_O_2_/Fe^2+^: fixed and 90 min reaction time).

The Fenton process efficiency for COD, TOC, TSS, and color removal as a function of solution pH was investigated (Figure 1). According to Figure 1, at low pH, the COD, TOC, TSS, and color removal efficiency are low, and with increasing pH up to 3, removal efficiency increased. After this point, COD, TOC, TSS, and color removal were decreased. 

The solution pH plays an important role in Fenton process. Figure 1 is evident that with increasing the initial pH from 1 to 3, the removal efficiency quickly increases and at pH > 3 decreases. The results from this experiment are in line with those studies reported by the researchers [6, 23, 26, 27]. They found that pH near 3 is usually optimum for Fenton oxidation. The low efficiency of Fenton process at pH < 3 is due to the formation of [Fe(II)(H_2_O)_6_]^2+^ complex, which reacts more slowly with H_2_O_2_ than [Fe(II)(OH)(H_2_O)_5_]^+^ and therefore produces lower OH radical [28]. Also, if the solution pH is too high, the iron precipitates as Fe(OH)_3_ and H_2_O_2_ decompose to oxygen and that will reduce its concentration in the solution [29, 30].

### 3.2. Effect of Reaction Time

**Figure 2:**
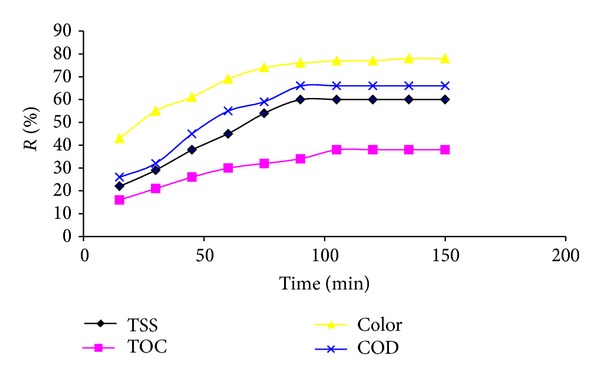
Effect of reaction time in Fenton process on COD, TOC, TSS, and color removal (H_2_O_2_/Fe^2+^: fixed and pH: 3).

The effect of reaction time on COD, TOC, TSS, and color removal during Fenton process is presented in Figure 2. The result showed that with rising reaction time, the COD, TOC, TSS, and color removal increased. The equilibrium time for COD, TOC, TSS, and color removal was obtained, 105 min. After the equilibrium time, the COD, TOC, TSS, and color removal did not change significantly.

Reaction time is an important factor in Fenton process. Different reaction time was reported in various studies. The reaction time for Fenton process in various studies has been fluctuated between 30 min and 3 h [6, 23, 27]. In some studies, based on electro-Fenton process, optimum reaction time has been reported less than 30 min [1].

### 3.3. Effect of Fe^2+^ Concentration

**Figure 3:**
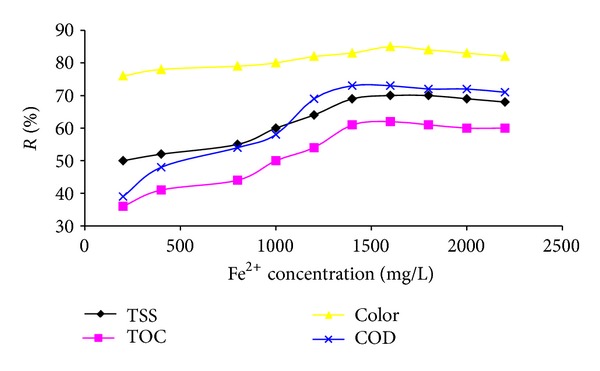
Effect of Fe^2+^ concentration on COD, TOC, TSS, and color removal (H_2_O_2_: 2500 mg/L, pH: 3, and 105 min reaction time).

Figure 3 shows the effect of Fe^2+^ concentration on the COD, TOC, TSS, and color removal during Fenton process. It can be seen that the Fenton efficiency of COD, TOC, TSS, and color removal increases with augmenting Fe^2+^ concentration. The optimum Fe^2+^ concentration for maximum COD, TOC, TSS, and color removal was 1.6 g/L. Further increase of Fe^2+^ concentration results in decrease in COD, TOC, TSS, and color removal efficiency. 

Based on operational costs and organic material removal efficiency, dosage of Fenton reagents will be determined. Generally, removal of organic matters improves with increasing concentration of iron salt. However, the removal increment may be marginal when the concentration of iron salt is high. Many studies have revealed that the use of a much higher concentration of Fe^2+^ could lead to the self-inhibition of OH radical by Fe^2+^ ions and decreasing the degradation rate of pollutants: ^•^OH + Fe^2+^→ Fe^3+^ + OH^−^ [30]. 

### 3.4. Effect of H_2_O_2_ Dose

**Figure 4:**
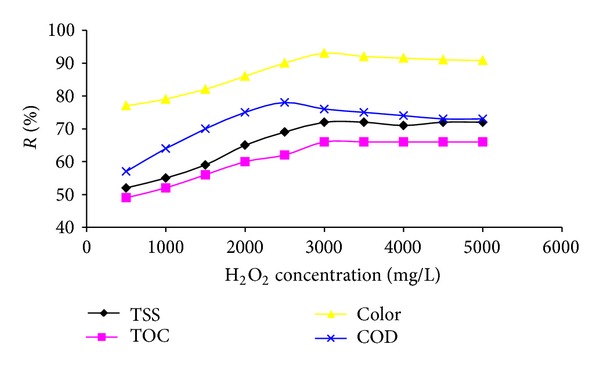
Influence of H_2_O_2_ dose on COD, TOC, TSS, and color removal (Fe^2+^: 1800 mg/L, pH: 3, and 105 min reaction time).

The influence of H_2_O_2_ dose on COD, TOC, TSS, and color removal is shown in Figure 4. The results showed that removal efficiency of COD, TOC, TSS, and color gradually increases as the H_2_O_2_ concentration is fluctuated from 500 mg/L to 3000 mg/L and then slowly decreased. It was observed that maximum COD, TOC, TSS, and color removal were obtained at H_2_O_2_ concentration of 3000 mg/L.

Landfill leachate is composed of a complex mixture of organic matter. During the oxidation process, more decomposition of organic matter causes more pollutant removal. This continues until formation of the end byproducts of oxidation reactions that mainly are short chain organic acids and are difficult to be further oxidized [13]. The presence of H_2_O_2_ in a high quantity can act as a scavenger for the OH radicals, thus reducing the kinetic rate of Fenton process [31]. In addition, due to decomposition of H_2_O_2_ and producing hydrogen gas, application of H_2_O_2_ more than the optimum value are caused flotation of generated iron sludge. Also, additional H_2_O_2_ causes problems in downstream processes and will prevent wastewater biological treatment [3, 32].

### 3.5. Effect of Fe^2+^/H_2_O_2_ Molar Ratio

**Figure 5:**
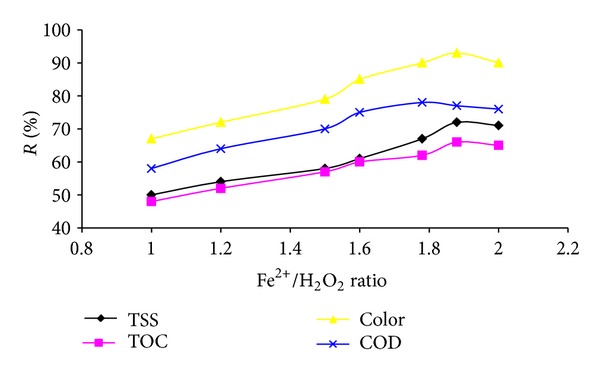
Effect of Fe^2+^/H_2_O_2_ molar ratio on COD, TOC, TSS, and color removal (pH: 3 and 105 min reaction time).

The effect of Fe^2+^/H_2_O_2_ molar ratio on the COD, TOC, TSS, and color removal with different Fe^2+^/H_2_O_2_ molar ratios within a range of 1 to 2 is presented in Figure 5. It can be seen that COD, TOC, TSS, and color removal increase with the rise in Fe^2+^/H_2_O_2_ molar ratio up to 1.88 and after this ratio decrease. 

In Fenton process, iron and hydrogen peroxide are two major chemicals that determining operation costs as well as efficiency. Determination of the favorable amount of Fenton's reagent is highly important. The results showed that removal efficiencies increased with the increase of H_2_O_2_/Fe^2+^ molar ratio, and further increase in H_2_O_2_/Fe^2+^ molar ratio produced less efficient improvement in removals. This fact is due to Fenton's reaction mechanisms proposed by other researchers [27]. If H_2_O_2_/Fe^2+^ molar ratio is low, the reaction rate follows second pseudoorder up to the stoichiometry ratio of 2Fe(II) ≅ H_2_O_2_. But, when the H_2_O_2_/Fe^2+^ molar ratio increases, the reaction kinetics approache toward zero order. However, at high H_2_O_2_/Fe^2+^ molar ratios, the mechanism changed, and the reaction became independent of hydrogen peroxide [29].

### 3.6. Kinetic Study of Leachate Treatment by Fenton Process

Reaction rates in the reactor must be specially determined to complete description and design of a reactor system and its direct effect on reactor size. Therefore, the study of reaction kinetics to prediction of pollutant removal rates is very important in designing and modeling of treatment process [33]. 

Determination of the kinetics of the Fenton on COD, TSS, TOC, and color removal reaction is needed to estimate the time required for COD, TSS, TOC, and color removal. A kinetic analysis was conducted by fitting the time-course performance data with zero-, first-, and pseudo-second-order kinetic equations as shown in Table 2.

**Figure 6:**
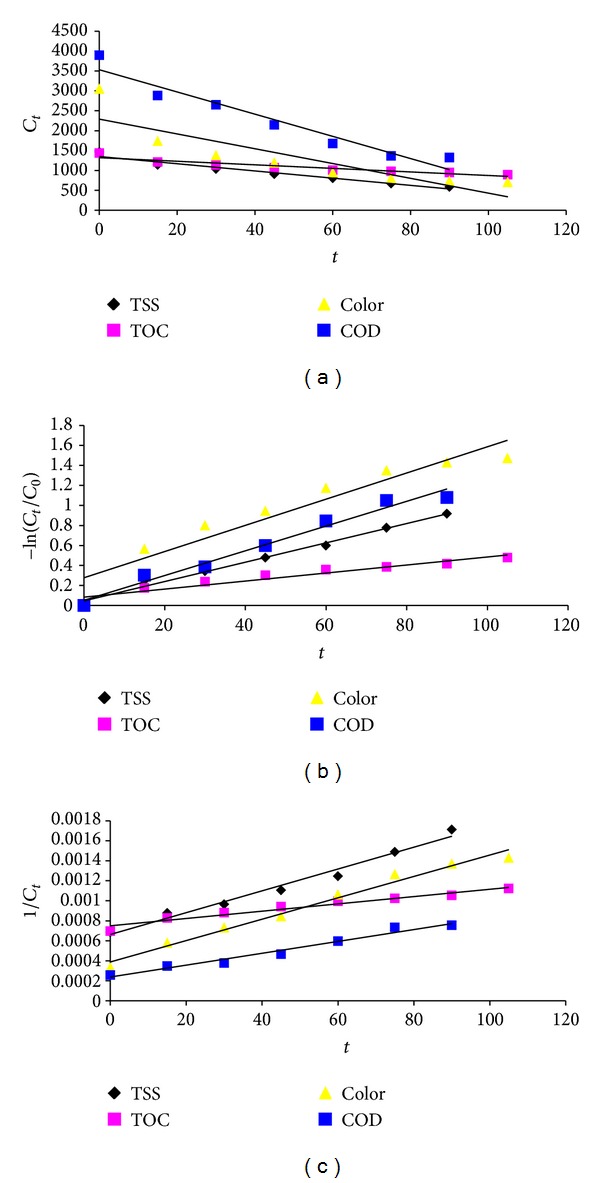
The results of kinetic study: zero-order (a), first-order (b), and second-order (c).

Where *r*_*c*_ is the rate of conversion, *k*_0_, *k*_1_, and *k*_2_ are reaction rate coefficients, *t* is time, and *C*_0_ and *C* are the initial and final concentration of the constituent in the liquid, respectively. The data were appropriately explained with first-order kinetic model (higher *R* ^2^), that presented that the model can successfully simulate COD, TSS, TOC, and color removal in the Fenton process. This results show high relationship between COD, TSS, TOC, and color removal efficiency and its initial concentration. In studies carried out by Guedes et al. and Yasar et al, in removal of synthetic dye by Fenton process, reaction rate is described with first-order kinetic model [17, 34]. The effects of COD, TSS, TOC, and color removal in the Fenton process are shown in Figure 6.

According to Figure 6, the linear relation for each pollutant removal confirms the fact that the kinetics of COD, TSS, TOC and color removal adherents of the exponential law with time.

Results showed that application of Fenton process is inadequate for landfill leachate treatment of Kermanshah city, but leachate characteristics could be greatly improved by Fenton process. The most important role of Fenton process is removing organic and toxic organic compounds and increasing the degradability of resistant organic compounds. Thus this process can be used as pretreatment for biological treatment. The main disadvantage of the Fenton process is that it requires disposal of excess sludge production.

## Figures and Tables

**Table 1:** Main characteristics of Kermanshah leachate.

Parameter	Value	Unit
Color	Dark brown	—
BOD_5_	800	mg/L
COD	3895 ± 180	mg/L
TOC	1438 ± 95	mg/L
TSS	1460 ± 80	mg/L
Alkalinity	2154 ± 130	mg/L
Color	3045 ± 45	Pt-Co
pH	7.8 ± 0.1	—

**Table 2:** Equations and linear forms and results of kinetics model.

Kinetic model	Equation	Linear form	Parameter	COD	TOC	TSS	Color
Zero-order	$r_{c}=\frac{dC}{dt}=k_{0}$	*C* − *C*_0_ = −*k*_0_ *t*	*K*_0_	27.93	4.48	9.04	18.58
			*R* ^2^	0.93	0.88	0.94	0.76
First-order	$r_{c}=\frac{dC}{dt}=k_{1}C$	$\ln\frac{C}{C_{0}}=-k_{1}t$	*K*_1_	0.012	0.004	0.009	0.013
			*R* ^2^	0.98	0.92	0.99	0.91
Second-order	$r_{c}=\frac{dC}{dt}=k_{2}C^{2}$	$\frac{1}{C}-\frac{1}{C_{0}}=k_{2}t$	*K*_2_	6 × 10^−6^	4 × 10^−6^	1 × 10^−5^	1 × 10^−5^
			*R* ^2^	0.97	0.91	0.95	0.94
